# Editorial: Employee resilience

**DOI:** 10.3389/fpsyg.2025.1631257

**Published:** 2025-06-06

**Authors:** José Alberto Martínez-González, Carmen D. Álvarez-Albelo, Urszula Kobylińska

**Affiliations:** ^1^Departamento de Dirección de Empresas e Historia Económica, Cátedra de Turismo CajaCanarias-Ashotel-ULL, Instituto Universitario de Desarrollo Regional and Instituto Universitario de la Empresa, Universidad de La Laguna, San Cristóbal de La Laguna, Spain; ^2^Departamento de Economía, Contabilidad y Finanzas, Cátedra de Turismo CajaCanarias-Ashotel-ULL, Instituto Universitario de Investigación Social y Turismo and Instituto Universitario de la Empresa, Universidad de La Laguna, San Cristóbal de La Laguna, Spain; ^3^Faculty of Engineering Management, Bialystok University of Technology, Bialystok, Poland

**Keywords:** Research Topic, employee resilience, concept, antecedents, outcomes

According to Kuntz et al. (2016), employee resilience is defined as “the capacity of employees to utilize resources in order to continually adapt and flourish at work, even when faced with adversity” (p. 460). This construct has been acknowledged as a cornerstone of individual and organizational performance. Still, several authors have pointed out that research on this topic requires further efforts to find a more unified conceptualization (e.g., Britt et al., 2016; Hillmann, 2021).

Identifying the factors that explain employee resilience is of utmost importance, since greater resilience positively impacts both individual wellbeing and organizational well-functioning. The literature has identified these factors and categorized them into individual, team, and organizational levels. The individual level includes factors such as positive coping, adaptation, realistic optimism, self-efficacy, and emotional intelligence (e.g., Luthans et al., 2007; Sharma and Tiwari, 2023). Regarding the team level, collective resources, cohesive structure, adaptability, learning orientation, positive relationships, adherence to norms, and reliable leadership can promote resilience (e.g., Hartwig et al., 2020; Li and Zhang, 2022). Finally, organizational values and culture, supportive and servant leadership styles, and resource access are crucial factors at the organizational level (e.g., Tvedt et al., 2023; Cai et al., 2024).

After about three decades of research, a broad consensus has been reached around three main findings. First, more resilient employees have better performance, satisfaction, and engagement (e.g., Kašpárková et al., 2018). Second, resilience protects against stress, reducing burnout and turnover intentions (e.g., Tonkin et al., 2018; West et al., 2020). Third, interventions designed to enhance what Luthans et al. (2007) defined as Psychological Capital (PsyCap), involving the psychological resources of hope, efficacy, resilience, and optimism, increase resilience at the workplace (e.g., Kuntz et al., 2016; Donaldson et al., 2019).

The Research Topic “*Employee resilience*” has aimed to improve our understanding of this concept, its antecedents, and outcomes. The six articles published here have contributed significantly to this triple aim.

Galy et al. offer a new conceptualization of resilience at work involving a multilevel, dynamic conceptual approach that encompasses the connection between individual, team, and organizational factors. Their theoretical approach can serve as a basis for future empirical research.

Yang et al. study work and family boundaries and their impact on employee resilience. This issue is critical nowadays with the development of TICs and the emergence of remote working. It has been concluded that firms should respect family boundary flexibility, consider it in career development planning, and establish policies to achieve it.

Gojny-Zbierowska studies the transfer of PsyCap from leaders to employees. She finds that developing employees' perceptions of organizational justice (POJ) improves their PsyCap, and leaders with higher PsyCap can compensate for POJ deficiencies. Moreover, this compensation turns out to be greater for older leaders.

Chen et al. investigate how and why collectivism-oriented human resource management (C-HRM) fosters employee resilience in the Chinese culture. Their findings indicate that C-HRM increases employees' perception of fairness, which improves their group identification and mutual respect.

Hao et al. study the conflicting impacts of shared leadership (SLP) on employee resilience. On the one hand, SLP enhances motivation and resilience. On the other, it can lead to employee role overload, which erodes resilience. In this context, organizational goal clarity is crucial, as it amplifies the positive effect of SLP and reduces the negative impact resulting from role overload.

Finally, Hu et al. study how employees' strategic goal sight (ESGS) affects their strategic actions. The authors find that ESGS positively influences employee strategic actions, with perceived insider status mediating in this relationship. However, greater openness to personal experience reduces the positive impact of ESGS on perceived insider status and strategic actions. These results can guide managers in enhancing employees' identification with the organization's strategic goals, thus enabling their achievement.
